# Abnormal Schwannoma-like Growth of multiple, multifocal BRAF V600E-positive Glioblastoma in the Interior Acoustic Canal with Leptomeningeal Infiltration: a case report

**DOI:** 10.1186/s13256-022-03272-3

**Published:** 2022-02-08

**Authors:** Roopa Jayarama-Naidu, Evelyn Gallus

**Affiliations:** 1 Department Internal Medicine, Kantonsspital Frauenfeld, Spital Thurgau AG, 8501 Frauenfeld, Switzerland; 2 Department Radiology, Kantonsspital Frauenfeld, Spital Thurgau AG, 8501 Frauenfeld, Switzerland

**Keywords:** Epithelioid glioblastoma, Neuroradiology, Multiple glioblastoma, Neuro-oncology, Metastasized glioblastoma, Case report, BRAF

## Abstract

**Background:**

Glioblastoma belongs to the most common and most aggressive tumor entity of the central nervous system with a poor prognosis of only few months. Once manifested, it grows fast and diffusely by infiltrating the surrounding brain parenchyma. Despite its aggressive behavior, glioblastoma rarely presents with multiple lesions and metastasis to intra- and extracranial tissues. Therefore, metastasized, multiple glioblastoma is limited to case reports. Our case describes an atypical primary bilateral manifestation of BRAF V600E-positive epithelioid glioblastoma with rapid metastasis and meningeosis glioblastoma while under adjuvant chemoradiotherapy.

**Case presentation:**

A 60-year-old Caucasian male patient presented with a seizure and numbness in his left arm. He was diagnosed with an abnormal primary bilateral manifestation of multiple, multifocal BRAF V600E-positive and isocitrate dehydrogenase (IDH) wild-type intracranial epithelioid glioblastoma with *O*^6^-methylguanine-DNA methyltransferase methylation (MGMT) at 12%. While being under the adjuvant chemoradiotherapy with temozolomide, the patient developed left-sided facial nerve weakness and hearing loss, dysarthria, and severe gait instability. Cranial magnetic resonance imaging showed that glioblastoma lesions advanced rapidly with a schwannoma-like growth pattern by invading the left internal acoustic meatus, adjacent cranial nerves, and leptomeninges. A lumbar puncture confirmed meningeosis glioblastoma. Four months after the initial diagnosis of glioblastoma, the patient died from the complications of the fast and diffuse metastasis.

**Conclusions:**

Glioblastoma rarely presents with metastases despite its aggressive and rapidly growing nature. Our case should increase awareness of symptom tracking in patients with glioblastoma to intervene early and efficiently. Moreover, refractory therapies for glioblastoma should underline the importance of personalized medicine.

## Background

Glioblastoma (GB) is among the most common as well as most aggressive tumors of the central nervous system, and has a poor prognosis [1–4]. The incidence in the European Union and North America is 2–3/100,000 per year with slightly higher incidence in men. The highest rate of new diagnosis occurs in late adulthood at a median age of 64 years but it can also occur in children at any age [5–7]. Predominantly, GB appears as a unilateral, solitary lesion, whereas primary multiple, especially bilateral, lesions are rare [8–13]. Likewise, cases of GB with schwannoma-like growth are exceptional [14–16]. The spreading of GB presumably occurs via the cerebrospinal fluid to the ventricular cavity with successive dissemination throughout the ventricular system and cerebrospinal leptomeninges [13]. Interestingly, intracranial GB infiltrating leptomeninges and causing meningeosis glioblastoma *per se* is rare [8, 17–19]. Metastasis of GB to the surrounding and contralateral brain parenchyma and to the extracranial tissue, with common sites being lungs, pleura, bones, bone marrow, skin, and cervical lymph nodes, has been observed [20–28]. The prevalence of extracranial metastasis is around 0.5%. However, metastases are more common in patients with recurrent disease than in patients at initial diagnosis [10, 13, 29–32]. Although recent research has introduced promising molecularly targeted compounds, one of the standard treatments utilizes temozolomide with simultaneous radiotherapy [33–39].

## Case presentation

**Fig. 1:**
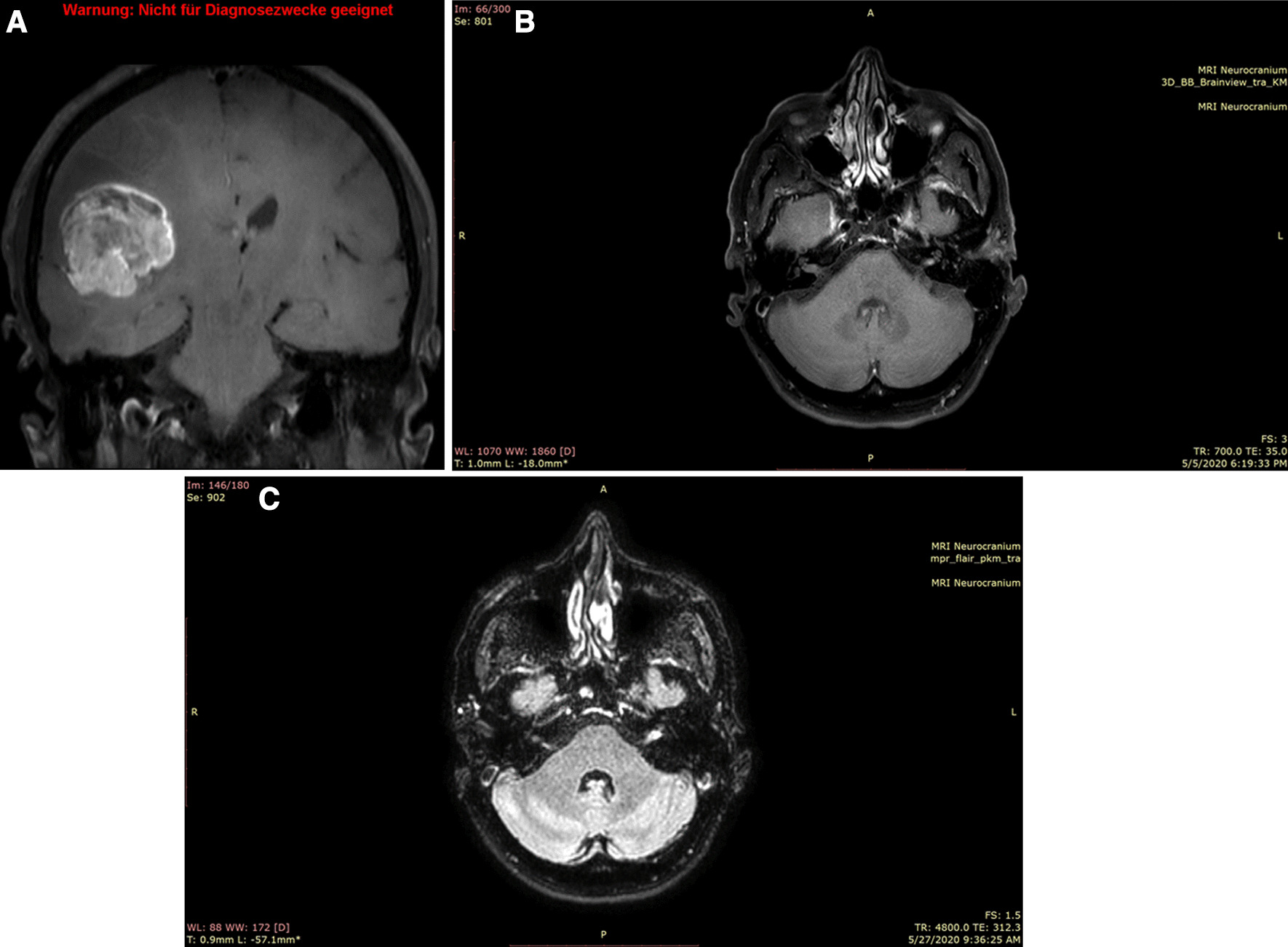
Brain magnetic resonance imaging at diagnosis and after resection of the tumor in the right temporoparietal lobe. **a** T1-weighted black-blood cMRI with contrast in transverse plane showing a tumor in the temporoparietal lobe at diagnosis (baseline). **b** T1-weighted cMRI after contrast showing a singular hyperintensity in the left internal acoustic channel at diagnosis (baseline). **c** Fluid-attenuated inversion recovery (FLAIR) image after contrast showing the lesion in the left internal acoustic channel after surgical resection of the right temporoparietal tumor

**Fig. 2:**
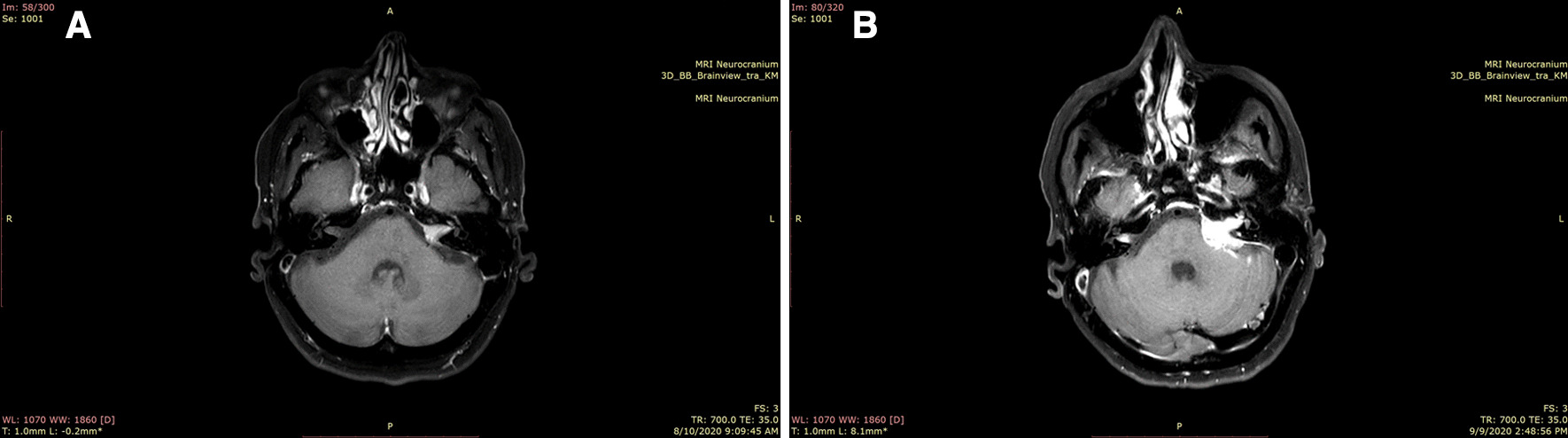
Brain magnetic resonance imaging of lesions in the left internal acoustic channel advancing with schwannoma-like growth 2 and 4 months after diagnosis. **a** T1-weighted cranial magnetic resonance imaging after contrast showing the tumor in the left internal acoustic channel at 2 months post-surgical follow-up. **b** T1-weighted cranial magnetic resonance imaging after contrast revealing infiltrative tumor growth with suspicious leptomeningeal involvement in the cerebellopontine angle within 4 months after diagnosis. Right nasal fracture upon fall is shown

**Fig. 3:**
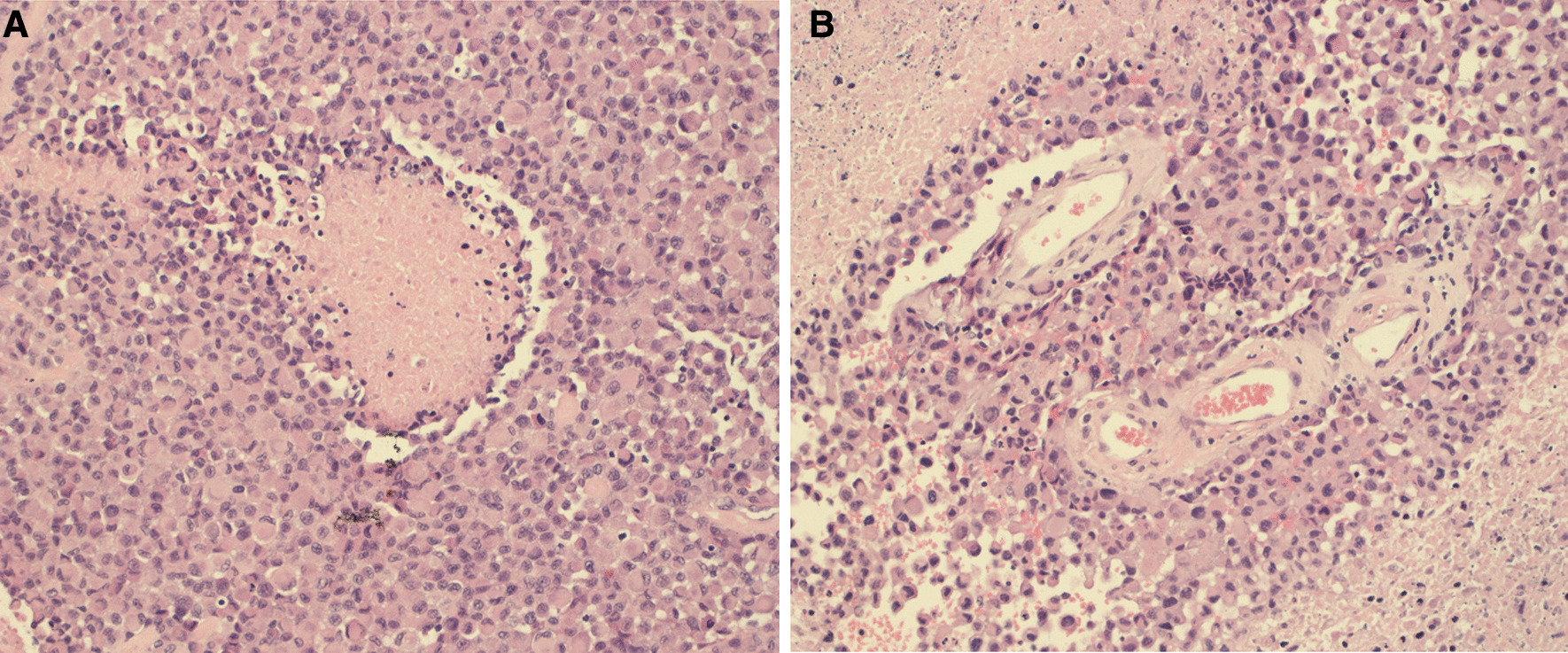
Histopathological analyses of the resected glioblastoma. **a** Histopathological specimen showing nuclear pseudopalisading, which is defined as the aggregation of tumor cells around the periphery of the necrotic areas, increased mitotic activity, and vascular proliferation. Pseudopalisading necrosis and vascular proliferation are the two important hallmarks of glioblastoma [55]. Magnification, 20×. **b** Histopathological specimen depicting an accumulation of viable tumor cells encircling the blood vessels in a large necrotic focus. The image also shows endothelial multilayering as a result of endothelial hyperplasia. These changes are mostly driven by vascular endothelial growth factor secreted by the tumor in response to hypoxia. Magnification, 20×

A 60-year-old Caucasian male was admitted to the emergency unit upon having a seizure, with no significant medical history. He reported a 2-month history of numbness in the left hand and intermittent dysarthria. Physical examination showed impaired fine motor skills and hypoesthesia in the left arm. Cranial magnetic resonance imaging (cMRI) revealed a multifocal 38 × 42 × 38 mm lesion in the right temporal lobe (Fig. 1a) and a singular lesion in the left internal auditory canal (IAC) with a discreet hyperintense signal and abnormal enhancement (Fig. 1b). Gross resection of the lesion in the right temporal lobe was performed. Immunohistopathological analyses identified the lesion as an isocitrate dehydrogenase (IDH) wild-type epithelioid glioblastoma with *O*^6^-methylguanine-DNA methyltransferase (MGMT) methylation at 12% and BRAF V600E mutation (Fig. 3). The patient was started on adjuvant concomitant chemoradiotherapy that included temozolomide [75 mg/m^2^ body surface area (BSA), d1–d42] and stereotactic radiotherapy (60 Gy split in 30 units) of the tumor cavity in the right temporal lobe and its marginalizing solid components [33]. The enhancement in the left IAC (Fig 1c) was not irradiated as the signal alteration was not interpreted as a metastasis [33]. To assess the therapy outcome, a cMRI was done on therapy day 42. The cMRI showed that the right-sided tumor cavity, including its solid components, remained unchanged in size but with a larger perifocal edema that was presumably a postradiogenic effect. However, the lesion in the left IAC excluded in the irradiation field was progressive (Fig. 2a). The oligoprogression prompted us to continue with temozolomide treatment at 100 mg/m^2^ BSA as maintenance therapy. Within 2 weeks, the patient was seen in the outpatient oncology clinic with a marked imbalance, as well as a new, rapidly advancing left-sided facial nerve weakness, dysphagia, dysarthria, and left-sided deafness albeit without lower central nervous dysfunction. The Romberg test was positive, and his gait was wide and ataxic, with assistance required to prevent falling during tandem walking trials. These symptoms were consistent with the lesion in the left IAC. Due to the fast deterioration and a fall leading to a nose bone fracture, we admitted the patient to our clinic. Owing to the persistent dysphagia, we decided to implant a percutaneous endoscopic gastrostomy (PEG) tube to avoid aspiration and malnutrition. Four weeks following the adjuvant chemoradiotherapy, the cMRI demonstrated a rapid growth of the lesion in the left IAC. This lesion measured 31 × 24 × 33 mm and infiltrated the adjacent structure, that is, the cranial nerves (II, V, VII–XII), the leptomeninges, and the left parotid gland (Figs. 2b, 3). In addition, the meninges of the Sylvian fissure showed an enhanced contrast uptake that breached the left orbit and cerebellum with suspicious infiltrations into the medulla oblongata. Moreover, the tumor cavity with its solid residues in the right temporal lobe was accompanied by an expanding edema (figure not shown). A lumbar puncture was performed and confirmed meningeosis glioblastoma on cytopathological analysis. Laboratory tests showed that hematological and organ functions were not impaired. To control the impact of the expanding intracranial mass, we initiated radiotherapy of the whole brain. As the patient deteriorated fast, we could neither start the patient on second-line therapy, such as antiangiogenic drugs or BRAF V600E inhibitors, nor recruit him in a clinical trial. The approval of BRAF inhibitors for treating V600E-mutated epithelioid GB was pending at the time (Swissmedic National Authorization for Drugs, cited September 2020); it would therefore have been an experimental approach. We decided to dispense further diagnostics and did not perform a biopsy of the left intrameatal lesion. At the request of the family and the patient, we focused on palliative care. The patient died 4 months after the initial diagnosis owing to the rapid tumor progression that led to paralyses of multiple cranial nerves. The family did not wish for an autopsy.

## Discussion

Multifocal GB is a highly aggressive and fast-growing tumor entity known for its poor prognosis and fatal complications. Typically, GB manifests as a single lesion, whereas multiple and particularly contralateral lesions are limited to only a few case reports [9, 10, 12]. One might suspect the short prognosis does not allow sufficient time for metastases to become clinically evident. In addition to the intracranial metastases of GB, intramedullary spinal metastasis, leptomeningeosis glioblastoma, and extracranial metastases are also very uncommon [8, 28, 31, 40–43]. Several mechanisms for metastasis have been postulated, including vascular invasion, perineural spreading, and direct invasion via the lymphatics [13].

In our case report, we describe a rare, abnormal primary bilateral manifestation of multiple, multifocal BRAF V600E-positive intracranial epithelioid GB lesions in both hemispheres that subsequently advanced rapidly and invaded the left internal acoustic meatus with perineural infiltration of the adjacent cranial nerves and leptomeninges while the adjuvant chemoradiotherapy was ongoing. In the continuous clinical assessments, the patient developed symptoms that resembled those of a benign schwannoma, which is a slow-growing and noninvasive tumor of the peripheral nervous system. To date, only a handful of clinical reports of primary glioma lesions mimicking a schwannoma have been reported [15]. However, the patient’s poor performance did not permit a biopsy of the intrameatal lesion to examine its etiology. As the baseline cMRI disclosed abnormal signal alterations in the left meatus, we cannot exclude metastatic growth. Therefore, we might assume that the intrameatal lesion was a metastasis of epithelioid GB. Moreover, its fast growth over a few weeks attests to the typical tumor biology of GB.

Moreover, the radiological findings implied an infiltration of the leptomeninges that might have caused a meningeosis GB. Recent literature describes the occurrence of meningeosis GB in patients with spinal metastases [41, 44–49]. In our patient, we cannot exclude spinal metastases since we did not obtain a scan of the spinal cord owing to the rapid deterioration of the patient. However, the patient did not experience neurological symptoms that were typical of spinal metastases such as paralyses, radicular pain, or peripheral sensory impairment. Clinical signs of meningitis were too vague to draw any conclusions.

Recommendations for the clinical management of highly progressive and metastasized GB are scarce, making palliative care the remaining option [1]. Radiotherapy is the preferred choice to control intracranial mass effect and to improve neurological symptoms, while second-line chemotherapy shows no survival benefit. Surgical intervention is necessary if compression increases the intracranial pressure [30, 41, 48]. With precision medicine becoming the state of the art, several promising molecular targeting therapeutics are under investigation. For example, the role of driver mutations, such as BRAF and its effect on pathogenesis of CNS tumors, has recently gained special interest [39]. In classic GB, BRAF mutations are rare, while the prevalence is higher in epithelioid GB (prevalence 1–2% versus 50%, *n* = 1320 samples) [50].

With the evolving era of personalized medicine, Kaley *et al.* identified the BRAF mutation as a promising druggable molecular target in CNS tumors by conducting the basket trial VE-BASKET [51]. BRAF mutation is known to negatively influence the overall prognosis in several tumor indications, for example, malignant melanoma, papillary thyroid cancer, and so on [52]. Hence, GB harboring a BRAF V600E mutation might exhibit a different, more invasive tumor biology than that of a BRAF wild-type GB. In our case, the V600E-positive epithelioid GB was also refractory to therapy as tumor progression occurred during combined chemoradiotherapy [33]. Here, the intratumoral heterogeneity might explain the treatment resistance of GB [53]. Certainly, cases of resistance to therapy should also encourage precision medicine research to establish novel algorithms for the treatment of GB [39, 50, 51, 54].

Currently, the data on the effect of BRAF inhibitors (BRAFi) on BRAF V600E-positive brain tumors are limited to a few experimental studies and case reports; thus, there is a high demand for further investigation. So far, the treatment of different types of brain tumors with BRAFi prolonged survival by several months to several years [37, 54]. Undoubtedly, response rates depend on the type of CNS tumor and tumor load [34–36, 38].

## Conclusion

Though our case is a rare observation, multiple metastases can lead to lethal tumor progression within days to weeks. Our study highlights several take-home messages: firstly, the clinician should focus on symptom tracking in patients with GB, so that symptoms that cannot be explained by the primary GB manifestation are recognized earlier. With these basic clinical assessments, an intervention can be planned efficiently. Symptom tracking might be extended with selected disciplines such as otolaryngology, neurology, and ophthalmology. Secondly, the future development of personalized cancer medicine should focus on molecular signatures, thereby introducing potential druggable targets. Besides molecular targeting compounds, immunotherapies are highly promising options, that is, T-cell therapies [chimeric antigen receptor T cells (CAR-T), tumor-infiltrating lymphocytes (TILS) and bispecific T-cell engagers (BiTEs)]. In particular, cases of refractory therapies necessitate the development of novel therapeutic algorithms. Finally, yet importantly, our case alludes to rare cases and their radiological presentation, thus improving the diagnostic workup overall. Should the left-sided lesion be regarded as a potential metastasis, a different therapy approach should be planned, for example, whole-brain rather than stereotactic radiotherapy.

## Data Availability

Yes.

## Abbreviations

GB: Glioblastoma
Gy: Gray
IAC: Internal acoustic channel
BRAFi: BRAF inhibitor
BSA: Body surface area
BRAF: Rapidly accelerated fibrosarcoma isoform B
BiTEs: Bispecific T-cell engagers
CAR-T: Chimeric antigen receptor T cells
cMRI: Cranial magnetic resonance imaging
PEG: Percutaneous endoscopic gastrostomy
FLAIR: Fluid-attenuated inversion recovery
TILS: Tumor-infiltrating lymphocytes
